# Comprehensive Analysis of Treatment Approaches for Lumbar Disc Herniation: A Systematic Review

**DOI:** 10.7759/cureus.67899

**Published:** 2024-08-27

**Authors:** Plamen Penchev, Ilko G Ilyov, Todor Todorov, Petar-Preslav Petrov, Petar Traykov

**Affiliations:** 1 Medicine, Medical University of Plovdiv, Plovdiv, BGR; 2 Medicine, Medical Univesity of Plovdiv, Plovdiv, BGR; 3 Anatomy, Histology, and Embryology, Medical University of Plovdiv, Plovdiv, BGR; 4 Thoracic Surgery, Evangelisches Krankenhaus Duisburg-Nord, Duisburg, DEU

**Keywords:** leg pain, low back pain, lumbar disc herniation management, conservative treatment, epidural injections, sciatica, exercises, physiotherapy, lumbar disc herniation surgery, lumbar disc herniation

## Abstract

Lumbar disc herniation is a common disorder that has an important impact on the quality of life and daily activities of those affected. It is defined as the displacement of the nucleus pulposus beyond the intervertebral space. This systematic review aims to evaluate and compare the efficacy of several treatment modalities, including conservative, pharmaceutical, and surgical interventions. The data sources utilized were PubMed, Google Scholar, Embase, and Cochrane. We conducted a systematic review of English-language articles published between 2019 and 2024, using the PRISMA guidelines. A total of 720 studies were identified during the search. Following the evaluation of the title, abstract, and full text, and the application of exclusion criteria, a total of 15 studies met the requirements for inclusion in the analysis. The results indicate that although conservative treatment is frequently successful in providing immediate relief of symptoms, surgical interventions may be required for patients experiencing neurological deficits or those who do not respond to conservative treatments. One limitation of this systematic review is the inclusion of a limited number of studies, which may affect the generalizability of the findings. Additionally, the review was restricted to English-language publications from 2019 to 2024, potentially excluding relevant research published in other languages or outside this timeframe.

## Introduction and background

Disc herniation is the displacement of the nucleus pulposus from the intervertebral space, causing back pain. The pain may extend to the lower limbs, be intense or sharp, and be accompanied by alterations in sensation or muscle weakness [1]. Disc herniation occurs when the nucleus pulposus protrudes through the annulus fibrosus, a dense collagenous ring that encircles the nucleus pulposus. As patients get older, the nucleus pulposus undergoes a degenerative process, leading to the most prevalent cause of symptom deterioration. Disc herniation can also occur due to congenital defects, connective tissue diseases, or trauma. Due to biomechanical factors, it is more common for this to happen in the lumbar and cervical spines, while the thoracic spine has a lower occurrence rate [1,2].

Lumbar disc herniation has a relatively high prevalence, occurring in 5 to 20 out of every 1000 people each year. The prevalence of LDH is highest between the ages of 30 and 50, with a ratio of 2 males to 1 female. Lumbar disc herniation can result in symptoms such as low back pain, leg pain, numbness, limited trunk flexion, muscle weakness, and instability. MRI is the most reliable method for confirming a lumbar disc herniation (LDH). Due to its exceptional soft-tissue visualization capabilities, this method is the most sensitive for visualizing a herniated disc, with a diagnosis accuracy of 97% [3].

The majority of symptomatic LDHs are of brief duration and typically disappear within a period of six to eight weeks. As a result, the initial approach to managing LDH is usually conservative with NSAIDs (or opioid analgesics if there is no effect from NSAIDs) and physical therapy, unless there are warning signs indicating the presence of urgent conditions such as progressive neurological impairment or cauda equina syndrome [3,4]. Surgical intervention is recommended for patients who continue to experience severe symptoms that are not alleviated by conservative and physical therapies or who have a neurological deficit/cauda equina syndrome. Performing surgery within a period of six months to a year in a patient with symptoms that require surgical intervention is associated with a quicker recovery and superior long-term outcomes [2-4].

The aim of this systematic review is to evaluate and compare the effectiveness of different treatment approaches for lumbar disc herniation, including conservative, pharmacological, and surgical options, to determine the most effective strategies for symptom relief and improved patient outcomes.

## Review

Methods

This systematic review used the Preferred Reporting Items for Systematic Review and Meta-Analysis (PRISMA) 2020 guidelines [5]. 

Search Sources and Strategy 

Table 1 presents the various sources and the search strategy used for selecting the papers for this review.

**Table 1:** Search strategy and keywords used in the resulting number of papers

Concept	Keywords and Medical Subject Headings (MeSH)	PubMed (700)	Google Scholar (10)	Embase (8)	Cochrane (2)
Lumbar Disc Herniation	lumbar disc herniation, intervertebral disc herniation	300 ✓	4 ✓	3 ✓	1 ✓
Sciatica	sciatica, radicular pain, sciatic nerve pain	150 ✓	1 ✓	2 ✓	0
Lumbar Disc Herniation Management	management of lumbar disc herniation, treatment of intervertebral disc herniation	90 ✓	1 ✓	1 ✓	0
Lumbar Disc Herniation Physiotherapy	physiotherapy for lumbar disc herniation, physical therapy for intervertebral disc herniation	60 ✓	1 ✓	1 ✓	1 ✓
Lumbar Disc Herniation Conservative vs. Surgical Treatment	conservative treatment for lumbar disc herniation, surgical treatment for intervertebral disc herniation	55 ✓	1 ✓	1 ✓	0
Epidural Injections	epidural injections, epidural steroid injections, lumbar epidural injections	30 ✓	1 ✓	0	0
Lumbar Disc Herniation Exercises	exercises for lumbar disc herniation, rehabilitation exercises for intervertebral disc herniation	15 ✓	1 ✓	0	0

Inclusion Criteria

Table 2 presents the various inclusion criteria used to select the papers suitable for this study.

**Table 2:** Inclusion criteria

Criteria	Details
Population	Adults aged 18 and older diagnosed with lumbar disc herniation by imaging (MRI, CT scan)
Intervention/Exposure	Studies examining treatment approaches, including both surgical (e.g., discectomy, laminectomy, microdiscectomy) and non-surgical treatments (e.g., physiotherapy, spinal injections, pharmacological treatments)
Comparison	Studies comparing different treatment approaches, including surgical vs. non-surgical treatments
Outcomes	Clinical outcomes such as pain relief, quality of life, functional improvement
Study Type	Original articles and case reports
Publication Date	Studies published in the last 5 years (2019-2024)
Language	Studies published in English

Exclusion Criteria

Table 3 presents the various exclusion criteria used to filter out the papers unsuitable for this study.

**Table 3:** Exclusion criteria

Criteria	Details
Population	Patients with cervical or thoracic herniations, or with significant co-morbid spinal conditions (e.g., tumors, fractures)
Intervention/Exposure	Studies not directly focused on lumbar disc herniation (e.g., general back pain management)
Comparison	Studies that do not report relevant clinical outcomes, such as those focused solely on imaging results without clinical correlations
Outcomes	Studies with incomplete or missing outcome data, or those focusing on outcomes not relevant to lumbar disc herniation
Study Type	Letter to the editor, meta-analyses, narrative reviews, systematic reviews, commentaries, non-peer-reviewed articles, conference abstracts, dissertations, duplicate studies
Publication Date	Studies published before 2019
Language	Non-English studies

Selection Process

We excluded any articles that did not have complete text available. Prior to proceeding, all 720 articles were evaluated exclusively based on their titles to ensure that none of the articles without full text available were of significant relevance to the study. This greatly reduced the limitations of the study. Subsequently, we imported the selected articles into Endnote and eliminated any duplicate papers. Every article underwent screening based on its titles and abstracts. The shortlisted papers underwent a comprehensive evaluation of their whole text, and only the relevant papers were examined. Only articles that satisfied the particular criteria for inclusion and exclusion were chosen for the process of shortlisting.

Quality Assessment of the Study

The reduced number of articles underwent quality checks using the appropriate quality appraisal tools. Randomized control trials were assessed using the Cochrane risk of bias assessment tool; clinical trials were assessed using the ROBINS-I tool (ROBINS-I tool (Risk Of Bias In Non-randomized Studies - of Interventions by Cochrane Methods, London, UK). 

Data Collection Process

Once the articles had been finished and extracted for the systematic review, the primary outcomes were evaluated together with any other essential data. We gathered information regarding the study's design, objective, and findings. 

Results

Study Identification and Selection

**Figure 1:**
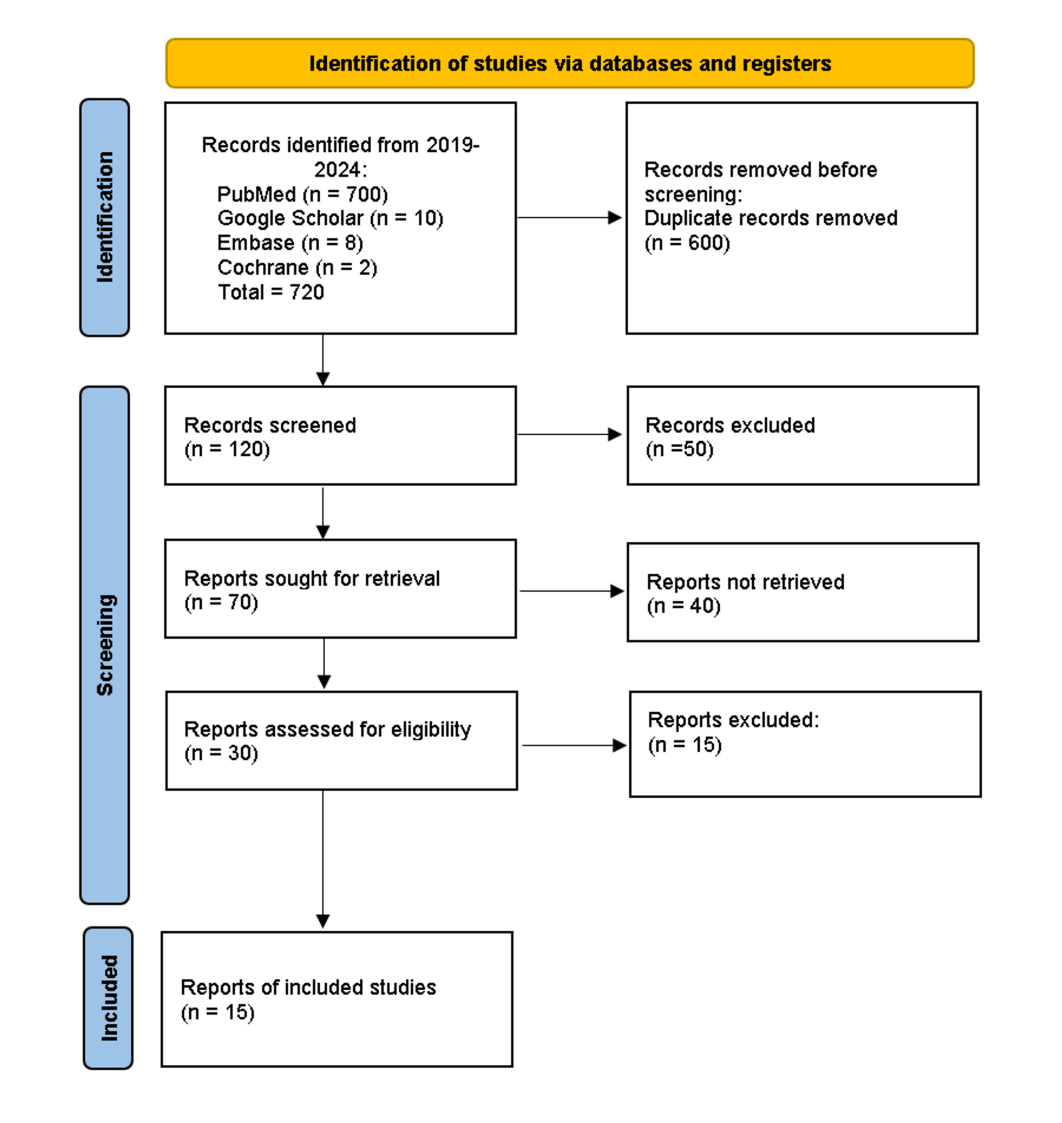
PRISMA flowchart showing the process of article selection PRISMA: Preferred Reporting Items for Systematic Review and Meta-Analysis

A total of 720 relevant articles have been identified by searching all databases. Prior to doing a thorough screening, a total of 600 duplicate articles were eliminated. After conducting a screening process based on the examination of titles and abstracts, a total of 30 articles were selected for further investigation. An analysis of the citations found in the identified papers resulted in the development of more studies. After evaluating the full-text papers that were selected, 15 of them were chosen for review based on their eligibility and quality. The method of selecting the studies is illustrated in Figure 1.

Studies Characteristics 

Detailed analyses of outcomes measured in the studies are shown in Table 4.

**Table 4:** Summary and characteristics of all the included studies

Serial No	Author and Year	Study Type	Aim of the Study	Number of Participants	Results	Conclusion
1	Gaowgzeh RAM et al., 2020 [6]	Randomized Controlled Trial	Assess effectiveness of spinal decompression therapy (SDT) + core stabilization exercises (CSE) vs. core stabilization exercises alone in reducing pain and improving functional impairment in chronic lumbar disc prolapse.	31	Both groups improved. SDT with CSE group: Numerical rating scale (NRS)mean difference 4.75 (t=12.81, p ≤ 0.001), Modified Oswestry Questionnaire (mOQ) mean difference 45.13 (t=29.34, p ≤ 0.001). CSE group: NRS mean difference 2.60 (t=13.67, p ≤ 0.001), mOQ mean difference 27.67 (t=24.52, p ≤ 0.001).	SDT with CSE more effective than CSE alone in reducing pain and disability in chronic lumbar disc prolapse patients.
2	Taşpınar G. et al., 2022 [7]	Randomized Controlled Trial	Evaluate impact of clinical Pilates exercises (CPE) on pain, functional status, flexibility, endurance, and quality of life in lumbar disc herniation patients.	54	After 6 weeks, CPE group showed significantly greater improvements in pain reduction, ODI scores, flexibility, endurance, and QoL (p < 0.05).	Clinical Pilates exercise effectively reduced pain, improved functional capacity, flexibility, endurance, and partially enhanced quality of life.
3	Ma Z. et al., 2021 [8]	Randomized Controlled Trial	Investigate conservative treatment for large lumbar disc herniation and factors influencing resorption.	409	89 had surgery, 320 had non-surgical treatment. Non-surgical group showed improvement in JOA scores from 10.22 ± 3.84 to 24.88 ± 5.69, with 84.06% achieving excellent outcomes. Disc protrusion decreased from 70.08 ± 30.95% to 31.67 ± 24.42%.	Conservative treatment is ideal for large disc herniation if there is no increasing nerve damage or cauda equina syndrome. Greater protrusions increase resorption likelihood.
4	Hu C. et al., 2021 [9]	Case report	Report efficacy of conservative treatment for lumbar disc herniation without neurological deficits.	1	After 4 months of conservative treatment, the patient had no symptoms. MRI showed unchanged herniated discs but no new lesions.	Conservative treatment can lead to symptom regression in sequestered lumbar disc herniation. Effective for patients not needing surgery.
5	Oros M. et al., 2019 [10]	Randomized Controlled Trial	Evaluate the effect of oral steroids vs. L-lysine in acute sciatica due to lumbar disc herniation.	90	Pain improvement was similar between dexamethasone and 5 or 10 mL L-lysine aescinate groups. Lowest pain levels in 10 mL L-lysine aescinate group.	Dexamethasone or L-lysine aescinate reduces acute radiculopathy pain. Dexamethasone may be more effective but L-lysine aescinate is a safer option.
6	Yadav RI et al., 2019 [11]	Randomized Controlled Trial	Compare microendoscopic discectomy (MED) vs. open lumbar discectomy (OLD) in terms of ODI scores, complications, and recovery metrics.	60	MED had shorter surgical times, less blood loss, and shorter hospital stays. Both groups showed significant ODI improvement; MED was superior in recovery metrics.	MED is a more efficient and effective option compared to OLD for lumbar disc herniation.
7	Gadjradj PS et al., 2022 [12]	Randomized Controlled Trial	Evaluate effectiveness of percutaneous transforaminal endoscopic discectomy (PTED) vs. open microdiscectomy in reducing leg pain.	613	PTED showed lower visual analog scores (VAS) for leg pain (median 7.0) compared to open microdiscectomy (median 16.0). PTED also resulted in less blood loss, shorter hospital stays, and earlier mobilization.	PTED is comparable to open microdiscectomy in reducing leg pain and improving other outcomes.
8	Bailey CS et al., 2020 [13]	Randomized Controlled Trial	Compare conservative treatment vs. surgical intervention for chronic sciatica due to lumbar disc herniation.	790	Surgical group had lower leg pain intensity (2.8 vs. 5.2), better Oswestry Disability Index scores at 6 months.	Microdiscectomy was more effective than conservative therapy in reducing pain intensity and improving disability after 6 months.
9	Hadžić E. et al., 2021 [14]	Randomized Controlled Trial	Assess impact of preoperative symptom duration on surgical outcomes for one-level lumbar disc herniation.	67	Surgery within the first 6 months resulted in significant reductions in radicular pain, sciatica bothersomeness, and disability (p < 0.001).	Early surgery within 6 months of symptom onset is preferable for better outcomes in radicular pain and sciatica bothersomeness.
10	Yavuz AY et al., 2022 [15]	Clinical Trial	Examine sleep quality and correlation between treatment modalities for lumbar disc herniation.	249	Early surgical treatment led to a 69% improvement in VAS and 63.8% in PSQI scores, compared to 28.5% and 38.6% improvements with extended conservative treatment.	Early surgical intervention is more effective in preserving sleep quality and preventing sleep loss compared to other modalities.
11	Uysal E. et al., 2023 [16]	Randomized Controlled Trial	Evaluate the effect of early vs. delayed rehabilitation on low back pain and quality of life post-microdiscectomy.	204	Early rehabilitation (2nd-week walk/waist exercise) showed better VAS scores and ODI at 1 month and 12 months compared to no-exercise group (p < 0.001).	Early introduction of physical activity is optimal for pain relief and faster recovery post-surgery. Regular exercise, even if started later, aids recovery.
12	Bilgin E. et al., 2021 [17]	Randomized Controlled Trial	Compare efficacy of pregabalin, betamethasone, and ibuprofen for postoperative pain management in single-level lumbar disc herniation.	60	Betamethasone significantly reduced visual analog scale (VAS) scores for back and leg pain during the first 24 hours and at 1 month compared to pregabalin and ibuprofen.	Betamethasone was more effective in reducing postoperative pain compared to pregabalin and ibuprofen.
13	Yörükoğlu HU et al., 2021 [18]	Randomized Controlled Trial	Determine the impact of erector spinae block on postoperative pain management in lumbar disc herniation surgery.	54	ESP group used 57% less morphine in 24 hours post-surgery compared to control group (11.3 mg vs. 27 mg). NRS scores were similar between groups.	Erector spinae block effectively reduces postoperative pain and morphine consumption in lumbar disc herniation surgery.
14	Wongjarupong A. et al., 2023 [19]	Randomized Controlled Trial	Evaluate the effectiveness of platelet-rich plasma vs. triamcinolone for single-level lumbar disc herniation.	30	PRP injections led to significant reductions in LegVAS scores at 6, 12, and 24 weeks, and in ODI scores at 24 weeks.	PRP treatment was more effective than triamcinolone for single-level lumbar disc herniation, with no adverse events reported.
15	Cai J. et al., 2020 [20]	Randomized Controlled Trial	Evaluate the effectiveness of epidural steroid injection (ESI) in reducing postoperative pain and complications in unilateral lumbar microdiscectomy.	90	ESI group had lower morphine consumption at 12, 24, and 48 hours post-surgery. No significant differences in pain scores, back pain, disability, or general health between ESI and placebo groups.	ESI reduced morphine usage but did not show notable differences in pain scores or complications compared to placebo after lumbar discectomy

Risk of Bias Assessment 

Table 5 presents the risk of bias assessment for the 13 randomized controlled trials (RCTs) included in the systematic review. The assessment was conducted using the Cochrane Risk-of-Bias Tool (Cochrane, London, UK), which evaluates potential biases across several domains. 

**Table 5:** Bias Assessment for Randomized Controlled Trials (RCTs)

No.	Study (Reference)	Study Title	Random Sequence Generation	Allocation Concealment	Blinding of Participants and Personnel	Blinding of Outcome Assessment	Incomplete Outcome Data	Selective Reporting	Other Bias	Overall Risk
1	Gaowgzeh RAM. et al. 2020 [6]	Effect of Spinal Decompression Therapy and Core Stabilization Exercises in Management of Lumbar Disc Prolapse	Low Risk	Low Risk	High Risk	Unclear Risk	Low Risk	Low Risk	Low Risk	High Risk
2	Taşpınar G. et al. 2022 [7]	The Effects of Pilates on Pain, Functionality, Quality of Life, Flexibility and Endurance in Lumbar Disc Herniation	Unclear Risk	High Risk	Unclear Risk	Low Risk	Low Risk	High Risk	Low Risk	High Risk
3	Ma Z. et al. 2021 [8]	Conservative Treatment for Giant Lumbar Disc Herniation: Clinical Study in 409 Cases	Low Risk	Low Risk	Low Risk	Low Risk	Low Risk	Low Risk	Low Risk	Low Risk
4	Oros M. et al. 2019 [10]	Steroids and L-Lysine Aescinate for Acute Radiculopathy Due to a Herniated Lumbar Disk	Low Risk	Low Risk	Unclear Risk	Unclear Risk	Low Risk	Low Risk	Low Risk	Unclear Risk
5	Yadav RI. et al. 2019 [11]	Comparison of the Effectiveness and Outcome of Microendoscopic and Open Discectomy in Patients with Lumbar Disc Herniation	High Risk	Unclear Risk	High Risk	High Risk	Low Risk	High Risk	Unclear Risk	High Risk
6	Gadjradj PS. et al. 2022 [12]	Full Endoscopic versus Open Discectomy for Sciatica: Randomised Controlled Non-Inferiority Trial	Unclear Risk	Unclear Risk	Unclear Risk	Unclear Risk	Low Risk	Low Risk	Low Risk	Unclear Risk
7	Bailey CS. et al. 2020 [13]	Surgery versus Conservative Care for Persistent Sciatica Lasting 4 to 12 Months	Low Risk	Low Risk	High Risk	Low Risk	Low Risk	Low Risk	Low Risk	Low Risk
8	Hadžić E. et al. 2021 [14]	Comparison of Early and Delayed Lumbar Disc Herniation Surgery and the Treatment Outcome	Low Risk	High Risk	Unclear Risk	Unclear Risk	Low Risk	High Risk	Low Risk	High Risk
9	Uysal E. et al. 2023 [16]	The Necessity and Timing of Exercise after Lumbar Disc Herniation Surgery	Unclear Risk	High Risk	High Risk	High Risk	High Risk	Unclear Risk	High Risk	High Risk
10	Bilgin E. et al. 2021 [17]	Post-Operative Pain Management for Single-Level Lumbar Disc Herniation Surgery: A Comparison of Betamethasone, Ibuprofen, and Pregabalin	Low Risk	Low Risk	Low Risk	Unclear Risk	Low Risk	Low Risk	Low Risk	Low Risk
11	Yörükoğlu HU. et al. 2021 [18]	Erector Spinae Block for Postoperative Pain Management in Lumbar Disc Hernia Repair	High Risk	High Risk	High Risk	High Risk	High Risk	High Risk	High Risk	High Risk
12	Wongjarupong A. et al. 2023 [19]	"Platelet-Rich Plasma" Epidural Injection: An Emerging Strategy in Lumbar Disc Herniation: A Randomized Controlled Trial	Low Risk	Unclear Risk	Unclear Risk	Unclear Risk	Low Risk	Low Risk	Low Risk	Unclear Risk
13	Cai J. et al. 2020 [20]	Efficacy and Safety of Epidural Steroid Injection Following Discectomy for Patients with Lumbar Disc Herniation: A Protocol	Low Risk	Low Risk	Low Risk	Low Risk	Low Risk	Low Risk	Low Risk	Low Risk

Table 6 provides the bias assessment for the clinical trial included in the systematic review. The ROBINS-I tool was used to evaluate the risk of bias across different domains specific to non-randomized studies. 

**Table 6:** Bias Assessment for the Clinical Trial

No.	Study (Reference)	Study Title	Confounding	Selection Bias	Classification of Interventions	Deviations from Intended Interventions	Missing Data	Measurement of Outcomes	Selection of the Reported Result	Overall Risk
1	Yavuz AY. et al. 2022 [15]	Treatment Method Selection for Sleep Quality Due to Lumbar Disc Herniation: Early Surgery or Others? A Single Center Clinical Trial	Low Risk	Low Risk	Low Risk	High Risk	Low Risk	Low Risk	Unclear Risk	Moderate Risk

Table 7 summarizes the general bias assessment for the case report included in the systematic review. Given the nature of case reports, the assessment is less structured but highlights key areas of potential bias.

**Table 7:** Bias Assessment for the Case Report

No.	Study (Reference)	Study Title	Reporting Bias	Selection Bias	Confounding Factors	Other Bias	Overall Risk
1	Hu C. et al. 2021 [9]	Spontaneous Regression of a Large Sequestered Lumbar Disc Herniation: A Case Report and Literature Review	High Risk	High Risk	High Risk	High Risk	High Risk

Discussion

This systematic study emphasizes the presence of more beneficial treatment choices for lumbar disc herniations. The manuscript includes 15 papers, with 13 being randomized controlled trials, one clinical trial, and one being a case report. These studies provide several therapy strategies for managing lumbar disc herniation.

Conservative Treatment - First Option in Absence of Neurological Deficit and/or Cauda Equina Syndrome

Lumbar disk herniation (LDH) is the primary degenerative condition affecting the spine, occurring in around 2-3% of the population [21]. Nevertheless, a minor fraction of patients (less than 10%) require surgical intervention. Surgical intervention is recommended when there is a progressive and significant weakness of the lower extremities and/or the presence of cauda equina syndrome. When these factors are not present, the initial treatment should include conservative measures such as nonsteroidal anti-inflammatory drugs (NSAIDs) or narcotic medications, physical therapy, and/or epidural transforaminal injections. The 2024 recommendations from the World Federation of Neurosurgical Societies (WFNS) state that nonsteroidal anti-inflammatory drugs (NSAIDs) have a significant beneficial impact on reducing acute low back pain and leg pain resulting from lumbar disc herniation (LDH). Initial treatment should prioritize conservative approaches for patients who do not have neurological deficits or cauda equina syndrome [21].

Pharmacological Therapy

Acetaminophen is the recommended initial treatment for low back pain, regardless of how long it has been present. While it may not be as effective as other analgesic drugs for acute pain, its few adverse effects make it the preferred option [22].

Nonsteroidal anti-inflammatory drugs (NSAIDs) - Nonsteroidal anti-inflammatory drugs (NSAIDs) suppress the activity of cyclooxygenase-1 (COX-1) and cyclooxygenase-2 (COX-2) enzymes, leading to reduced inflammation and pain relief. These drugs are commonly used for discogenic pain [21]. Roelof et al. state that non-selective NSAIDs are more effective than placebo in alleviating low back pain without the requirement for further analgesics [23].

Thiocolchicoside is a preferred muscle relaxant due to its anti-inflammatory and analgesic effects. Carisoprodol, cyclobenzaprine, and metalaxone are alternative medications for treating acute low back pain. However, they may cause adverse effects such as gastrointestinal problems, dizziness, and headaches [21-23].

Non-Pharmacological Therapy

Physical therapy is crucial in the management of low back pain. Studies have demonstrated that performing back extension exercises and making behavioral posture changes will alleviate back pain caused by lumbar disc herniation (LDH). Yoga or tai-chi activities may achieve comparable outcomes [24].

Bed rest is the term used to describe the practice of restricting all physical activity for a period of more than two days. Altun et al conducted a retrospective cohort study involving 23 patients with LDH. They found that a period of 2 weeks of bed rest resulted in a reduction of low back pain caused by LDH [25].

Spinal decompression therapy (SDT) is a recently employed method for treating lumbar disc herniation (LDH). In a recent study conducted by Gaowgzeh RA et al, it was found that a 6-week combination of spinal decompression therapy (SDT) with core stabilization exercises (CSE) is more effective in reducing low back pain, leg discomfort, and disability in patients with lumbar disc herniation, compared to CSE alone [6].

In a recent randomized controlled trial conducted by Taşpınar G. et al, it was found that pilates exercises, which enhance core stabilization and motor control, effectively reduced pain levels at rest, general pain, and pain during movement, while also improving disability and quality of life. The trial included 54 patients, consisting of 30 females and 24 males, who were experiencing symptomatic LDH [7].

A recent study conducted by Ma Z et al. evaluated 409 patients who had conservative treatment. The results showed that 320 patients (78.24%) experienced symptom alleviation and recovered with this treatment. Among these 320 patients, 189 (59.06%) achieved complete resorption of the herniated disc [8]. Macki et al. conducted a study on 53 cases of large-type disc herniation that spontaneously regressed. They observed that the symptoms improved after a period of 1.33-1.34 months. Additionally, a follow-up MRI after 1 year showed the resorption of the herniated disc [26].

Hu C.et al recently reported an unusual case of symptom alleviation in a 32-year-old male patient who had been experiencing low back pain and weakness in his right leg for a duration of one week. These symptoms were caused by disc herniations at the L3/4, L4/5, and L5/S1 levels. Following a period of 4 months of conservative treatment, the patient did not exhibit any symptoms. The MRI imaging revealed complete regression of the herniated disc at the L4/L5 level, while no alterations were observed in the discs at the L3/L4 and L5/S1 levels [9]. Despite the presence of two herniated discs, this patient remains asymptomatic. This particular case demonstrates the need to make decisions on lumbar disc herniations based on symptoms rather than relying just on imaging.

A recent study conducted by Oros M et al. examined 90 patients with acute radiculopathy caused by lumbar disc herniation. The study found that administering IV dexamethasone or L-lysine aescinate resulted in pain improvement on the 15th and 30th day. However, if a long-term analgesic effect is required, dexamethasone may be the preferable option. L-lysine aescinate is a viable substitute for dexamethasone in alleviating pain symptoms, as it does not have the same adverse effects [10].

Surgical Indications and Recommendations

The latest 2024 WFNS recommendations [27] about the role of surgery in lumbar disc herniation state that (1) surgery should be adjusted to each individual case. (2) If the patient has severe motor deficit, progressive neurological dysfunction, or if conservative treatment has failed, surgery may be required. It is recommended to do surgery earlier in cases with lumbar disc herniation with significant motor impairment, as this is linked to quicker recovery and potential improvement in motor function. (3) While minimally invasive treatments offer certain short-term benefits, there is currently insufficient data to support or discourage the selection of a particular surgical procedure for lumbar disc herniation (LDH). (4) Sequestrectomy and routine microdiscectomy provide comparable clinical outcomes in terms of pain management, recurrence rate, functional outcome, and complications in the short to medium term. (5) Lumbar fusion is not advised as a standard treatment after initial discectomy in patients with isolated herniated lumbar discs that cause radiculopathy. Lumbar fusion is an option for patients with herniated discs who experience chronic axial back pain, have severe degenerative changes, or have instability associated with radiculopathy caused by herniated lumbar discs.

Minimally Invasive Surgery vs Open Surgery

Surgical intervention is indicated for lumbar disc herniation when conservative treatment becomes ineffective and/or the patient presents with neurological impairment or cauda equina syndrome. Traditionally, the standard surgical approach for treating lumbar disc herniation has been open discectomy. However, there has been a rising preference for minimally invasive techniques. Microendoscopic discectomy (MED) is a widely recognized and advancing procedure used to treat lumbar disc herniation. It has a success rate of around 90%. Both methods are equally efficient in alleviating radicular pain. However, MED offers advantages such as shorter hospital stays, reduced morbidity, less blood loss, decreased aesthetic exposure, and quicker intraoperative time compared to open discectomy [11]. However, percutaneous transforaminal endoscopic discectomy (PTED) is not inferior to open microdiscectomy in terms of leg pain reduction and functional status improvement, as both surgical methods show similar outcomes during long-term follow-up [12]. In a recent meta-analysis conducted by Gadjradj PS et al, 14 prospective trials were examined. The findings revealed that PTED is equally effective as open microdisectomy in terms of reducing leg pain and improving quality of life in the long term. There were no significant differences observed between the two procedures [28]. MED is a secure and efficient alternative to traditional open discectomy for patients with lumbar disc herniations. However, it should be noted that performing endoscopic methods correctly requires additional education and experience. 

Why Early Surgery Is Important?

Surgical intervention is the preferred treatment for persistent sciatica lasting between 4 to 12 months, which is caused by lumbar disc herniation [12,13]. This approach provides superior pain relief compared to conservative treatment, as evidenced by the 6-month follow-up. Nevertheless, a controlled trial has demonstrated that surgery provides superior treatment outcomes compared to conservative treatment for alleviating symptoms within the initial 6 months in individuals with lumbar disc herniation [13]. Patients who underwent a waiting period of 12 weeks or longer before undergoing surgery reported more severe pain at the 6-month postoperative stage compared to those who had a shorter waiting period [14]. Quon JA et al report that symptom duration of 6 months or more is linked to a more unfavorable outcome compared to a shorter period following either surgical or nonsurgical therapy [29]. According to Hadžić E. et al., performing surgery to treat lumbar disc herniation within the first 6 months of symptoms can be advantageous as it reduces the intensity of radicular pain, the bothersomeness of sciatica, and the disability experienced by the patient [14]. Lumbar disc herniation can significantly impair the sleep quality of patients, resulting in sleep deprivation, which can contribute to the development of social and psychological diseases. In their study, Yavuz AY et al. found that early surgical intervention was more effective than other treatment techniques in preserving sleep quality and preventing a reduction in sleep quality [15].

Postoperative Rehabilitation Should Be Initiated as Fast as Possible

Engaging in consistent physical activity is strongly advised for obtaining long-term relief and accelerating the recovery process following surgery, which is essential for sustaining a high quality of living [16]. It is advisable to start physical exercises promptly, and if there is a delay, standard back exercises may speed up the process of rehabilitation. The goal of postoperative rehabilitation and exercises after lumbar disc herniation surgery is to optimize the recovery rate and prevent muscle atrophy, by strengthening the lumbar muscles and ensuring spinal stability [16]. Pester et al. suggest that there should be no concern towards early mobility after surgery and emphasize its importance in the treatment of both acute and chronic back pain [30]. Oosterhuis et al conducted a systematic study which concluded that there should be no limitations on movement following lumbar disc herniation surgery, and physical exercises should commence between the 4th and 6th week [31]. 

Postoperative Pain Management

Dexamethasone, pregabalin, and ibuprofen are effective for postoperative analgesia in lumbar disc herniation surgery. Dexamethasone and ibuprofen exhibit superior efficacy during the initial phase in comparison to pregabalin. Nevertheless, the analgesic effects of all medications are nearly equivalent at the end of the first month [17]. Recent studies indicate that patients who received steroids experienced a significant increase of over 20% in their visual analog scale (VAS) ratings within a single day, and this impact lasted for more than 30 days [32]. Another study suggests that epidural steroid injections can result in a minor yet detectable reduction in VAS scores for leg pain, lasting between 2 to 6 weeks [33]. Kashefi P et al. concluded that NSAIDs exhibit superior efficacy and induce a lower risk of nausea and emesis. Appropriate administration of NSAIDs reduced the consumption of opioid drugs and reduced the adverse effects they had [34].

The erector spinae block (ESB) has been employed for postoperative pain management in patients who have undergone lumbar disc herniation surgery. A recent study conducted by Yörükoğlu HU. et al has shown that ESB provided efficient pain relief in patients who underwent surgery for lumbar disc herniation. The authors saw a substantial reduction in morphine consumption at 6, 12, and 24 hours following surgery, resulting in a total drop of 57% in morphine consumption 24 hours after the operation compared to the control group [18]. Singh et al. conducted a randomised controlled study including 40 patients, where they found that bilateral ESB effectively reduced morphine usage and improved patient satisfaction with postoperative analgesia [35].

Wongjarupong A et al. recently employed transforaminal epidural platelet-rich plasma (EPP) injections to provide postoperative pain relief for 15 patients who had undergone lumbar disc herniation surgery. The patients who had EPP injections demonstrated a statistically and clinically significant decrease in their leg score on the VAS at 6, 12, and 24 weeks. Additionally, there was a reduction in their Oswestry disability index (ODI) at 24 weeks [19]. A recent study has found that 20 patients observed a notable decrease in postoperative pain and improvement in disability for up to six months after receiving EPP injections [36]. In a recent study conducted by Cai J. et al., 90 patients were given transforaminal epidural steroid injections (ESI) for postoperative pain relief. The authors hypothesized that ESI would result in lower VAS scores, reduced morphine consumption at 12, 24, and 48 hours, shorter hospital stays, and improved quality of life [20].

Limitations

Our review is limited by the inclusion of only 15 studies, all published in English between 2019 and 2024, which may limit the generalizability of our findings and introduce publication bias. The focus on recent studies could exclude valuable earlier research. Additionally, the heterogeneity in study designs and patient populations complicates direct comparisons, potentially affecting our conclusions. Despite efforts to assess study quality, inherent biases like selection and reporting bias may still influence the results. Lastly, excluding non-peer-reviewed literature might have omitted emerging data relevant to lumbar disc herniation treatments.

## Conclusions

Lumbar disc herniation (LDH)-related back pain and leg pain is a prevalent global health issue. When cauda equina syndrome, motor or other neurological deficits are not present, it is recommended to start with conservative treatment as the initial approach for LDH. A combinatorial approach involving adjustments to activities, administration of medication, as well as execution of physical therapy provides favorable results in the majority of patients. Surgical intervention may be necessary for patients who do not respond to conservative treatment for a period of 6 weeks and continue to experience persistent or recurring clinical symptoms. The purpose of surgery is to relieve short-term symptoms, improve long-term outcomes, and improve overall quality of life. Prompt initiation of physical exercises is crucial for expediting the recovery process. 
